# 

**Published:** 1837-07-01

**Authors:** 


### MEDICO-CHIRURGICAL

### REVIEW,

AND

### JOURNAL

OF

### PRACTICAL MEDICINE.

*(NEW SERIES.)*

VOLUME TWENTY-SEVEN,

[1st of APRIL to 30th of SEPTEMBER,]

1837.

VOL. VII. of DECENNIAL SERIES.

Nothing to index

EDITED

BY JAMES JOHNSON, M.D.

PHYSICIAN EXTRAORDINARY TO THE LATE KING,

AND

HENRY JAMES JOHNSON, ESQ.

LECTURER ON ANATOMY AT THE SCHOOL OF ST. GEORGE'S HOSPITAL IN KINNERTON STREET.

Surgeon Gen'l's Office LIBRARY Washington, D.C. 57467

LONDON:

PUBLISHED BY S. HIGHLEY, 32, FLEET STREET,

*Re-printed in New York, by Mr. Wood.*

1837.

WI
ME 75 T

PRINTED BY F. HAYDEN,
Little College Street, Westminster.

### BIBLIOGRAPHICAL RECORD.

1. On Deformities of the Chest and Spine. Illustrated by Plates. By WILLIAM COULSON, M.R.C.S. &c. Second ed. greatly enlarged. Hurst, St. Paul's Church-yd. 1837.

2. Proceedings at the 18th Anniversary Meeting of the Hunterian Society, held on the 6th February 1837, with the Report, &c.

3. Historical and Medical Researches into the Origin, Nature, and Treatment of Syphilis. By M. DEVERGIE, Sen. Translated by JOHN SINCLAIR INNES, Surgeon. Duodecimo, Edinburgh, 1837.

4. The American Cyclopædia of Practical Medicine and Surgery, a Digest of Medical Literature. Edited by ISAAC HAYS, M.D. &c. Part X. Philadelphia, 1836. September. *Ascites to Azygos.*

5. Homœopathy briefly examined in a Letter to Sir Henry Halford, M.D. by a Licentiate. 8vo. pp. 66. Highley, 1837.

6. A Companion to the Ship's Medicine Chest. Being a Short Treatise on the Diseases of Seamen and Others in Tropical Climates, &c. By W. G. TADDY, Surgeon. Duodecimo. Highley, 1837.

☞ *A useful little work where there is no surgeon on board.*

7. The Anatomy, Particular and Surgical, of the Human Body, illustrated by a Series of Engravings, partly copied from the works of the most celebrated authors, and partly from Nature. By A. FYLE, F.R.S. &c. late Demonstrator of Anatomy in the University of Edinburgh. Corrected and arranged by P. D. HANDYSIDE, M.D. F.R. S.E. &c. 4to. Fasciculus 2. Coloured 4s. Edinburgh and London, 1837.

8. Oration delivered before the Members of the Royal Medical Society of Edinburgh, at the Celebration of their Centenary, February 13, 1837. By W. B. CARPENTER, Senior President of the Society. 8vo. pp. 36. Edinburgh, 1837.

9. Maladies des jeunes filles pendant d'Epoque de l'Accroissement. Premier Memoire, de la Chlorose. Par BUREAUD RIOFREY, M.D. pp. 34. Sherwood and Co. 1837.

10. Maladies des Organes de la Voix. Premier Memoire, Sur Laryngite Aigue. Par BUREAUD RIOFREY, M.D. Sherwood and Co. 1837.

11. Guy's Hospital Reports, No. IV. April 1837. Edited by GEORGE H. BARLOW, M.D. and JAMES P. BABINGTON, M.A. &c. 8vo. pp. 310. Highley, 1837.

12. A Treatise on Diseases of the Eye and its Appendages. By RICHARD MIDDLEMORE, M.R.C.S. Surgeon to the Birmingham Eye Infirmary, &c. In two volumes. 8vo. pp. 844. Longman, 1835.

13. The Works of John Hunter, F.B.C. with Notes. Edited by JAMES F. PALMER, Senior Surgeon of the St. George's and St. James's Dispensary, &c. In four volumes, illustrated by a volume of plates, in Quarto. Vol. the Second, pp. 448, with a Fascic. of Plates. Longman & Co. 1837.

14. A Practical Compendium of Diseases of the Skin, &c. By JONATHAN GREEN, M.D. Second Edition corrected and improved, with two illustrative plates. 8vo. pp. 371. Whittaker, 1837.

15. A Treatise on the Diagnosis and Treatment of Diseases of the Chest. Part the First, Diseases of the Lung and Windpipe. By WILLIAM STOKES, M.D. Physician to the Meath Hospital, &c. 8vo. pp. 557. Dublin, 1837.

16. The Continental and British Medical Review, &c. No. 3. May, 1, 1837.

17. A Dictionary of Practical Medicine, &c. &c. By JAMES COPLAND, M.D. F.R.S. &c. Part IV. London, May, 1837. Longman and Co.

18. An Address delivered to the Members of the Worcestershire Natural History Society on the Opening of the W. Museum, September 15, 1836. By CHARLES HASTINGS, M.D. 8vo. pp. 57. Sherwood. May, 1837.

19. The Oration delivered before the Medical Society of London, at their 64th Anniversary Meeting, March, 1837. By EDWARD HEADLAND, Honorary Secretary to the Society, &c.

20. A History of British Quadrupeds. By THOMAS BELL, F.R.S. Part IX. price 2s. 6d. May, 1837.

21. An Experimental Inquiry into the Comparative State of Urea in Healthy and Diseased Urine, and the Seat of the Formation of Sugar in Diabetes Mellitus; an Essay, &c. By ROBERT M'GREGOR, Licentiate of the Royal College of Surgeons, Glasgow, &c. Glasgow, 1836.

---

22. Clinique Medicale de l'Hôpital de la Charité; ou Exposition Statistique des diverses Maladies traitées à la Clinique de cet Hopital. Par J. BOUILLAUD, M.D. Professeur de Clinique, &c. In two volumes, 8vo. pp. 508-488. Bailliere, Regent-street. May, 1837.

---

23. Medical Essays. No. 2. The Imagination. By J. HUNGERFORD SEALY, M.D. 8vo. pp. 91. Sherwood, London, 1837.

---

24. The Principles of the Theory and Practice of Medicine, including a Third Edition of the Author's Work upon Diagnosis. By MARSHALL HALL, M.D. F.R.S. &c. &c. 8vo. pp. 503. Sherwood, 1837.

---

25. The Northern Flora, containing the Wild Plants of the North of Scotland. By ALEXANDER MURRAY. M.D. 8vo. pp. 150, with Appendix. Part the First. Edin. 1837.

---

26. A Treatise on the Diseases and Injuries of the Larynx and Trachea, founded on the Essay to which was adjudged the Jacksonian Prize for 1836. By FREDERICK RYLAND, Surgeon to the Town-Infirmary, Birmingham. 8vo. pp. 338, with Plates. Price 18s. Longman and Co. 1837.

---

27. Rudiments of Physiology. By Dr. FLETCHER. Part 3 on Life, as manifested in Sensation and in Thought. Edited by ROBERT LEWINS, M.D. with a Biographical Memoir of the Author. Edin. April, 1837.

---

28. The Philosophy of Human Nature, in its Physical, Intellectual, and Moral Relations, &c. &c. By HENRY M'CORMAC, M.D. 8vo. pp. 564. Longman, 1837.

---

29. Toxicological Table exhibiting the exact appearances on Analyzing any Solution suspected to contain the following Poisons, Salts of Copper—Salts of Arsenic Corrosive Sublimate—Oxalic Acid—Prussic Acid—Opium—Salts of Lead. By JOHN ROBINSON, M.D.

---

30. Two Lectures on Lithotrity and the Bi-lateral Operation, &c. By EDWARD LEE, Esq. Surgeon. From the London Medical Gazette.

31. Additional Remarks on the Use of English Mineral Springs, especially those of Bath, Cheltenham and Leamington, with the most recent Analyses. By ED. LEE, Esq. London, Churchill. June, 1837.

---

32. The Teeth a Test of Age, considered with reference to the Factory Children. By EDWARD SAUNDERS. 8vo. pp. 76. Renshaw, Strand, 1837.

---

33. *In. M.S. to be presented to the Royal Medico-Chirurgical Society.*

On the Philosophy of Blood-letting and Venous Congestion. By MARTIN PAINE, M.D. of New York.

☞ *The Society had just closed its sittings for the Session, when the above MS. was received. It will be presented in due time for the ensuing season.*

---

34. What Asylums were, are, and ought to be, being the Substance of Five Lectures delivered before the Managers of the Montrose Royal Lunatic Asylum. By W. A. F. BROWNE, Surgeon, &c. 8vo. pp. 231. Adam Black, Edinburgh, 1837.

---

35. The Transactions of the Provincial Medical and Surgical Association. Instituted in 1832. Vol. 5. Sherwood, 1837.

---

36. The Philosophy of Instinct and Reason. By J. STEPHENSON BUSHNAN, M.D. 8vo. pp. 316, with plates. Edinb. 1837.

---

37. Address of Earl Stanhope, President of the Medico-Botanical Society, for the Anniversary Meeting, January 16th, 1837.
☞ *Eloquent, comprehensive, yet minute.*

---

38. Aphorisms on Natural and Difficult Parturitions, Puerperal Diseases, and on the Physical Management of Infants. By MICHAEL RYAN, M.D. Duodecimo. pp. 28.

---

39. The Medico-Chirurgical Formulary; or a Conspectus of the Best Prescriptions, &c. &c. By MICHAEL RYAN, M.D. M R.C. Duodecimo, 1837.

---

40. An Introduction to Medical Botany, &c. &c. By THOMAS CASTLE, M.D. F.L.S. 8vo. pp. 276. Cox.

---

41. A Translation of the Pharmacopœia Londinensis, of 1837, with Descriptive and Explanatory Notes on the Materia Medica, &c. By THOMAS CASTLE, M.D. F.L.S. &c. Duodecimo, pp. 260. Cox, Borough, June, 1837. Price 5*s*.

